# Radiotherapy Combined With Androgen Deprivation for Bone Oligometastases After Primary Curative Radiotherapy for Prostate Cancer: A Retrospective Study

**DOI:** 10.1097/MD.0000000000002789

**Published:** 2016-02-12

**Authors:** Jun-Xin Wu, Li-Mei Lin, Jun-Yan He, Liang Hong, Jin-Luan Li

**Affiliations:** From the Department of Radiation Oncology, Teaching Hospital of Fujian Medical University, Fujian Provincial Key Laboratory of Translational Cancer Medicine, Fujian Provincial Key Laboratory of Tumor Biotherapy, Fujian Provincial Cancer Hospital, Fuzhou, China.

## Abstract

To evaluate the effects and toxicity of radiotherapy (RT) combined with androgen deprivation (AD) for bone oligometastases after primary curative RT for prostate cancer (PCa).

We retrospectively analyzed 30 consecutively treated PCa patients with bone oligometastases from April 2005 to July 2014. All patients underwent RT combined with AD for oligometastatic bones after curative RT for PCa. Measured outcomes included overall survival (OS) rate, local control (LC), progression-free survival (PFS), pain relief, and toxicities. Statistical analysis was performed with SPSS17.0.

The median follow-up was 32.5 months (range, 0.6–50.3). The 3-year PFS and OS rates were 22.8% (95% CI, 13.4–37.5%) and 69% (95% CI, 51.7–81.1%), respectively. The number of bone oligometastases and RT schedule were found to be significantly associated with OS on univariate analysis (*P* < 0.05, respectively). The 3-year OS for patients with 1 and >1 metastases was 78.8% versus 42.2%, respectively (*P* = 0.037). The long-course RT was associated with better 3-year OS compared with short-course (76.4% vs 44.1%, *P* = 0.03). A total of 15 (83.3%, 15/18) patients achieved pain relief. No grade 3 toxicity was observed.

Long-course RT combined with ADT was effective and well-tolerated in PCa patients with bone oligometastases after curative RT for PCa. Further randomized controlled trials are needed to corroborate the findings.

## INTRODUCTION

Prostate cancer (PCa) death is frequently ascribed to metastatic disease in most patients.^1^ Androgen deprivation therapy (ADT) has been the standard treatment for metastatic PCa after primary treatment in the past decade. However, deterioration in quality of life (QOL) during ADT has been widely reported, prompting the search for alternative therapies.^2,3^ In addition, effective local treatment may reduce the burden of systemic therapies and potentially improve the QOL. Nevertheless, limited PCa recurrence is rarely treated with aggressive treatment because of the high tendency toward metastatic dissemination and the advanced age of the patients.

The concept of oligometastases was first reported by Hellman and Weichselbaum.^4^ They hypothesized that the local treatment of patients with limited number of metastatic or recurrent lesions, using surgical resection and RT, improved systemic control. To date, the management of oligometastases has been highlighted in many tumors such as colorectal cancer, renal cell carcinoma, and lung cancer, resulting in long-term survival after excision of isolated lung or liver metastasis.^5–10^

Patients with oligometastatic PCa have better prognosis than those with extensive metastases, which is similar to many other solid tumors.^11,12^ This finding should be considered when managing oligometastases. Hellman and Weichselbaum also indicated that maintaining a localized form of PCa was an effective strategy to delay the progression of disease.^13^ Surgery has been reported in several studies to treat oligometastatic PCa with curative intent and improved disease-free survival.^14^ However, for patients who refused or were intolerant to surgery, external beam RT (EBRT) might be a good option.^15^

Although RT for PCa oligometastases has been reported in many retrospective studies,^16^ only limited results of RT in bone oligometastases have been published. Skeletal-related events (SREs) are the major factors underlying morbidity in PCa and result in bone pain and deterioration of QOL.^17^ A previous study showed that RT improved motor function in oligometastic patients with spinal cord compression.^18^ Therefore, careful management of bone oligometastases in PCa is required to prevent skeletal complications and prolong physical activity.

In our study, we evaluated the efficacy and toxicity of RT combined with AD for bone oligometastases after primary curative radiotherapy (RT) for PCa, and identified the prognostic factors distinguishing PFS and OS.

## MATERIALS AND METHODS

### Patients

This study was approved by the ethics committee of Fujian Provincial Cancer Hospital, in accordance with the Helsinki Declaration. A total of 30 consecutive PCa patients were retrospectively analyzed in this study. Patients underwent RT combined with AD for oligometastatic bones after curative RT for PCa in our institution, from April 2005 to July 2014. All patients had pathologically confirmed prostate carcinoma, life expectancy >3 months, Karnofsky Performance Status >70, ≤3 bone metastatic lesions detected on bone scans and F-18 choline PET/CT, primary curative RT for PCa, and no evidence of further visceral metastases. Pretreatment evaluation included a complete history, physical examination, complete laboratory tests, and staging. All bone oligometastases were independently diagnosed by a radiologist and nuclear medicine physician regarding the clinical history of the patients.

### Radiation Therapy

Depending on the depth of bone metastases, RT was delivered through a single posterior field or parallel opposed fields with 6 or 10 MV X-rays. The radiation regimen included short-course and long-course RT. The short-course RT was performed with a dose of 20 Gy in 5 fractions over 1 week (n = 2) or 30 Gy in 10 fractions over 2 weeks (n = 16). The long-course RT was 37.5 Gy in 15 fractions over 3 weeks (n = 3), 40 Gy in 20 fractions over 4 weeks (n = 5), and 50 Gy in 25 fractions over 5 weeks (n = 4). The biologic effectiveness of radiation schedule can be compared with the Equivalent Dose in 2-Gray Fractions {EQD2 = D × [(d + α/β)/(2 Gy + α/β)], D = total dose, d = dose per fraction}.^19^ Assuming an α/β of 3 y for PCa, the EQD2 of short-course RT for 20 Gy in 5 fractions over 1 week, and 30 Gy in 10 fractions over 2 weeks were 28.0 and 36.0 Gy, respectively. The EQD2 of the long-course RT for 37.5 Gy in 15 fractions over 3 weeks, 40 Gy in 20 fractions over 4 weeks, and 50 Gy in 25 fractions over 5 weeks were 41.3, 40.0, and 50.0 Gy, respectively.

### ADT

Neoadjuvant and concomitant ADT used bicalutamide and LH–RH analog (goserelin). The duration of ADT depended on age, tolerance, and PSA response.

### Follow-Up

The data of patients were retrospectively analyzed. All patients were followed up clinically every 3 months in the first year after treatment. Subsequently, the patients were regularly evaluated every 6 months in the second and third year, and annually thereafter. Local progression was defined as local recurrence in the previously treated region.

### Evaluation of Response

The primary endpoints of the study included overall survival (OS), progression-free survival (PFS), and local control (LC). The OS was defined as the time between the complication of RT and death from any cause and the PFS was defined as the interval between the complication of RT and the date of progression or death. LC was calculated from the complication of RT to the local recurrence. The potential prognostic factors were as follows: age (≤75 years vs >75 years), localization of bone metastases (spine vs pelvis/hip vs thigh-bone vs ribs), Gleason score of primary disease (≤6 vs >6), risk groups (low vs intermediate vs high), number of bone oligometastases (1 vs >1), initial treatment (RT ± ADT vs RT ± ADT + other treatments) and radiation schedule (short- vs long-course RT). Treatment-related adverse events were evaluated according to the Common Terminology Criteria for Adverse Events v3.0.^20^ Acute toxicity occurred during RT or within the first 3 months thereafter, and late toxicity was defined as an event occurring or lasting more than 3 months following treatment.

Pain relief after RT, prevention of spinal cord compression and pathological fracture were also analyzed. Pain relief response was classified as follows: “relief” when the daily dosage of the analgesic was decreased; “progressive” when the dosage was increased and “steady” when the dosage remained the same. Short-term pain relief occurred within 1 month after RT.

### Statistical Analysis

Survival curves were constructed using the Kaplan–Meier method and compared using the log-rank tests. All tests of significance were 2-sided: differences in *P* values of <0.05 were considered significant. Statistical analyses were performed using the SPSS statistical software package version 17.0.

## RESULTS

### Patient Characteristics

A total of 30 patients were included in the present study. The patient characteristics are shown in Table 1. The median age of the patients was 78 years (range, 50–87 years). Five (16.7%) patients were identified as low-grade, 11 (36.6%) intermediate-grade and 14 (46.7%) high-grade at diagnosis according to the National Comprehensive Cancer Network Guidelines. Of all the patients, 13 (43.3%) had only 1 bone lesion, 11 (36.7%) had 2 lesions, and the other 6 (20.0%) had 3 lesions, respectively. All patients received curative RT for PCa, while 9 patients underwent radical retropubic prostatectomy.

**TABLE 1:**
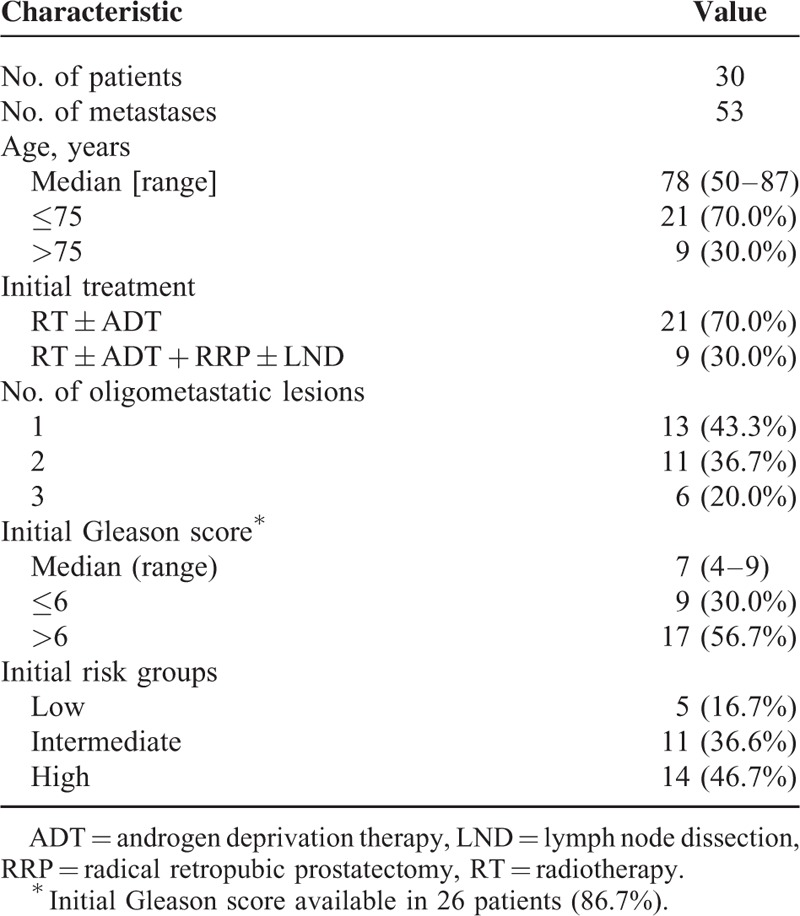
Patient Characteristics (N = 30)

### Treatment Characteristics

Table 2 displays the treatment profile. The EQD2 ranged from 28 to 50 Gy assuming an α/β-ratio of 3 Gy. Eighteen patients (60.0%) received RT for pain relief, 11 (36.7%) for prevention of SREs and the other 1 (3.3%) for spinal cord compression. Twenty-six patients (86.7%) received zoledronic acid and 5 (16.7%) received chemotherapy. Short-course RT was administered to 46.7% of the patients (14/30) and 53.3% (16/30) received long-course RT. Twenty-five (83.3%) patients received ADT for ≤12 months, and 5 patients were treated for >12 months. ADT was not interrupted in 4 patients because of multimetastatic progression and biochemical recurrence during treatment.

**TABLE 2:**
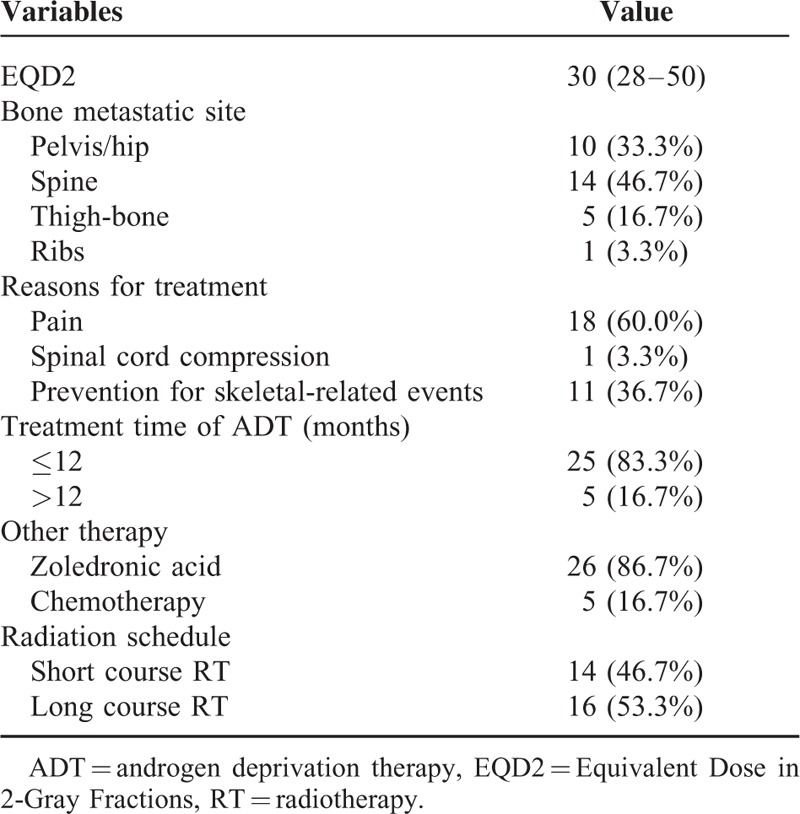
Treatment-Related Characteristics

### Survival and Local Control

The median follow-up period was 32.5 months (range, 0.6–50.3 months). Two patients were lost during follow-up and 8 patients died at the time of analysis. A total of 15 (50.0%) patients experienced progression, including 4 (13.3%) with local recurrence and 11 (36.7%) with new distant metastases. The local recurrence was due to in-field failure. The new distant metastases included lung (9.0%, 1/11) and out-field bone (91%, 10/11). The time to median progression was 8.8 months (range, 0.9–30.6 months). The time to median local and distant progression was 5.5 months (range, 2.0–46.2 months) and 8.8 months (range, 0.9–30.6 months), respectively. The 3-year PFS and OS rates were 22.8% (95% CI, 13.4–37.5%) and 69.0 (95% CI, 51.7–81.1%), respectively. The LC rates at 1, 2, and 3 years were 92%, 86%, and 75%. Of the 15 patients who developed progression, 10 (66.7%, 10/15) underwent local palliative RT with or without ADT, 2 (13.3%, 2/15) received chemotherapy and 3 patients (20.0%, 3/15) received no treatment. Irradiation was repeated with 30 Gy in 10 fractions (n = 8), 39 Gy in 13 fractions (n = 1) and 25 Gy in 10 fractions (n = 1). Relief of bone pain occurred in 60% (9 of 15) of all patients treated for progression, 8 after reirradiation and 1 after chemotherapy, respectively.

### Prognostic Factors Affecting OS and PFS

**FIGURE 1:**
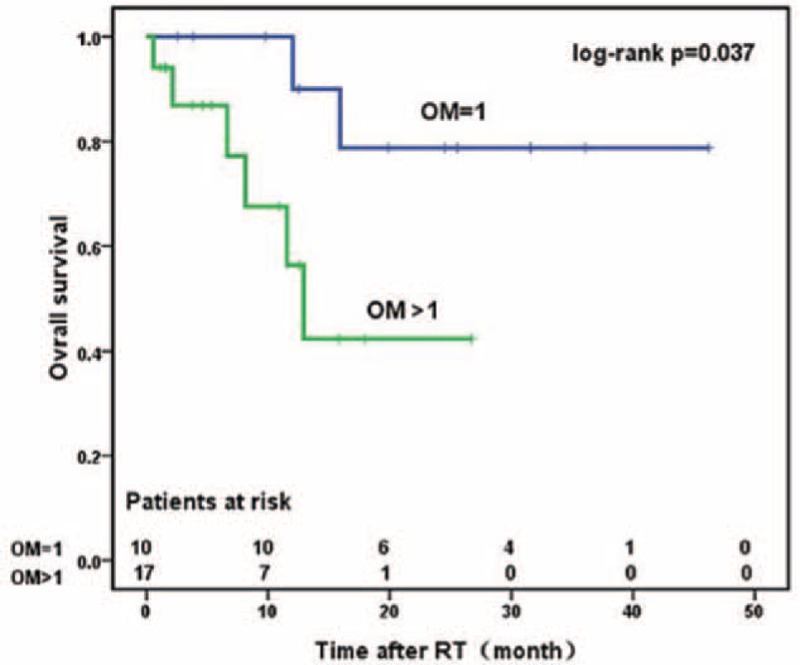
Overall survival of patients with OM = 1 and patients with OM > 1. OM = oligometastases, RT = radiotherapy.

**FIGURE 2:**
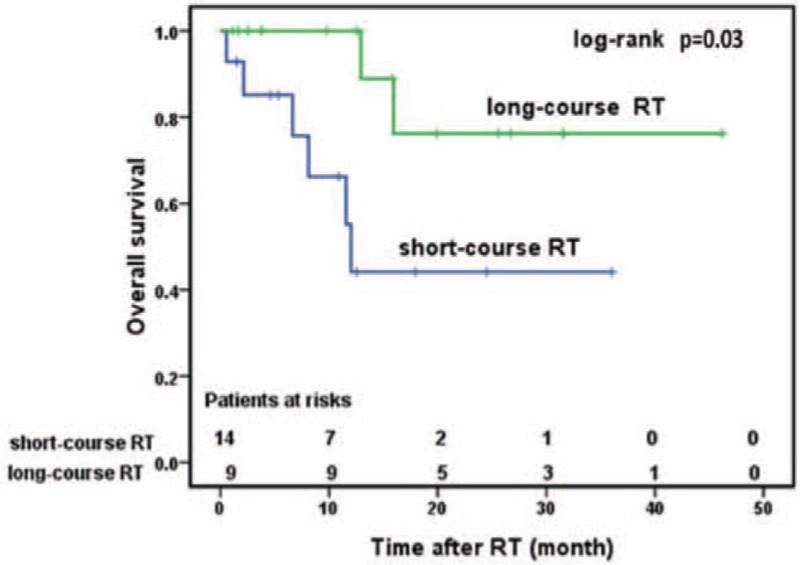
Overall survival of patients treated with short-course RT and patients treated with short-course RT. RT = radiotherapy.

Univariate analysis showed that the number of oligometastases and the RT procedures were significantly associated with OS (*P* < 0.05, respectively). The 3-year OS rate was 78.8% versus 42.2% for patients with 1 and >1 metastases after initial treatment, respectively (*P* = 0.037) (Figure 1). The 3-year OS was 76.4% and 44.1% for patients receiving long-course RT and short-course RT, respectively (*P* = 0.03) (Figure 2). None of the other variables was significant: age (*P* = 0.25), Gleason score (*P* = 0.11), initial treatment (*P* = 0.53), risk groups (*P* = 0.46), and localization of metastases (*P* = 0.82). Neither the number of oligometastases (*P* = 0.09) nor RT schedule (*P* = 0.625) was associated with PFS.

### Pain Relief and Tolerance

The treatment outcomes are shown in Table 3. Fifteen out of 18 patients (83.3%) with pain experienced relief within 1 month after RT. However, 5 of them experienced pain relapse within 12 months due to progression. Neither pathological fracture nor spinal cord compression was observed at the sites treated with RT. Two patients (6.6%) with spinal cord compression and 1 (3.3%) with pathological fracture were observed out-field. The RT treatment for bone oligometastases was well tolerated. We observed only 3 (10%) acute urinary events (2 with Grade 1 and 1 with Grade 2, respectively). Late toxicities were not found after RT or reirradiation for bone oligometastases at the last follow-up.

**TABLE 3:**
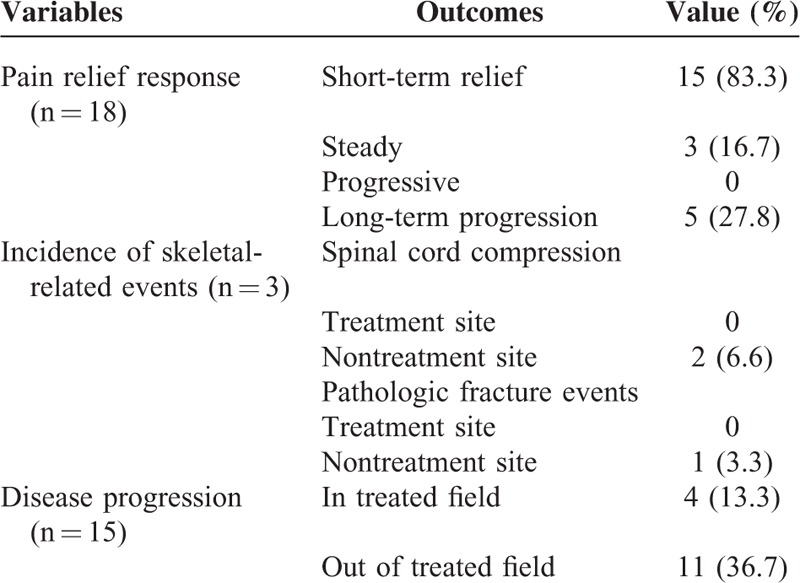
Treatment Outcomes

## DISCUSSION

This study provides the first report of efficacy and toxicity of RT combined with AD for bone oligometastases after primary curative RT for PCa. We found that the number of oligometastases and the RT schedule were associated with OS. Most patients with pain gained relief within 1 month after RT.

A few studies had previously concentrated on the quantifying risk according to the number of metastatic lesions in PCa.^21,22^ They had reported that the number of oligometastases might be an important prognosticator in PCa. Among these studies, Singh et al^23^ found that patients with <5 metastatic lesions had improved 5-year OS compared with those with ≥5 lesions (73% vs 45%) similar to metastasis-free patients. Similarly, Schick et al^15^ reported a 3-year biochemical recurrence-free survival of 66.5% in patients treated for only 1 metastatic lesion and 36.4% (*P* = 0.031) in those treated for >1 metastases. However, this factor was not confirmed in multivariate analysis. Our outcomes are similar to those findings showing that improved OS was significantly associated with the number of PCa metastatic lesions. In the present study, the 3-year OS rates were 78.8% (95% CI, 38.1–94.3%) for patients with only one metastatic lesion and 42.2% (95% CI, 11.9–70.5%) for patients with >1 metastatic lesions (*P* < 0.05), respectively. Therefore, PCa patients with oligometastases were recommended more aggressive treatment such as surgery or RT.

The 3-year OS of 69% in our study was lower when compared with the study published by Schick et al,^15^ in which the OS rate was 91.7% using conventional high-dose RT for oligometastases in PCa after 3 years of follow-up.^15^ Schick et al enrolled patients with other cancers. In addition, higher RT dose might also influence outcomes. Similarly, Tabata et al^24^ had shown that patients treated with conventional RT doses of >40 Gy had a superior survival compared with those treated with <40 Gy (90.5% vs 50.0%, *P* = 0.0116). However, after adjusting for other confounding variables, long-course RT was not an independent risk factor for OS in multivariate analysis.^24^ Our study was consistent with these findings, suggesting a relationship between radiation schedule and OS (*P* = 0.03). Additionally, the 3-year OS rates were 76.2% (95% CI, 33.2–93.5%) for patients treated with long-course RT and 44.1% (95% CI, 14.5–70.7%) for patients treated with short-course RT (*P* < 0.05), respectively. These results explain the higher EQD2 (>40 Gy for long-course RT vs <40 Gy for short-course). Therefore, long-course RT might be a better option for PCa bone oligometastases.

Schick et al^15^ reported 50 PCa patients treated with conventional RT for lung, lymph node and bone oligometastases, and achieved a better 3-year PFS than our study (58.6% vs 22.8%). The relatively poorer PFS can be partially explained by the fact that the bone metastases, which constitute only 31.5% in Schick's study but 100% in our study, might be more aggressive than lymph node oligometastases. Schick et al^15^ also showed an improved PFS with normalized total doses of >64 Gy versus <64 Gy in univariate analysis (65% vs 41.8%, *P* = 0.005), which was also confirmed in multivariate analysis (HR 0.37, *P* = 0.034). However, the dose–response effect was not related to better PFS in our study, probably due to the efficacy of ADT.

LC of oligometastases not only provide symptomatic relief but also reduce the burden of systemic therapy.^25^ Bone metastases, if not treated effectively, will cause spinal cord compressions and pathological fractures, which reduce QOL.^26^ Therefore, by limiting the local progression, successful local therapy might enhance the QOL in patients diagnosed with bone oligometastases. Using conventional RT, Rades et al^18^ recently showed a 3-yr LC of 66% in treating oligometastases, compared with 75% in our study. The improved LC might be attributed to the fact that the Rades et al's study enrolled other cancers such as lymphoma and lung cancer, which were more unfavorable than PCa. Although the 2 studies are not directly comparable, both showed that RT produced effective short-term LC of oligometastases.

As reported, the majority of metastatic PCa might be refractory to continuous AD after 2 to 3 years.^27^ Intermittent AD was a relatively effective strategy compared with continuous AD as demonstrated in a few Phase II–III trials.^28,29^ However, once the PSA started to increase after the first off-treatment period, restarting hormone therapy during intermittent AD remains controversial. In addition, the AD-free intervals described by many studies are not comparable. A prospective study by Yu et al^30^ focused on 100 PCa patients with biochemical relapse following retropubic prostatectomy or RT and reported a median off-treatment interval of 9.5 months. Schick et al^15^ reported, however, that a median off-ADT interval of 19 months after the RT combined with ADT for PCa oligometastases, was comparable with our study (17 months) and Bhandari's study (6–16 months).^31^

The Radiation Therapy Oncology Group 9714 study including 34% of PCa patients reported 40% of pain relapse after 1 year, which is comparable with our study (55.6%).^32^ However, the evaluation point of 3 months after RT for pain relief might not be suitable for PCa oligometastases because of the relatively improved survival. In our study, 15 out of 18 patients suffering from pain (83.3%) obtained relief. For patients without bone pain, prevention of pathological fracture and spinal cord compression was the main purpose. The study showed absence of any complications in the sites treated with RT. However, there were 3 out-field SREs, 2 with spinal cord compression, and another with pathological fracture. Therefore, the present study suggested that RT for bone metastases in PCa patients decreased the incidence of SREs.

Despite the limited number of patients in our study (30 patients with a total of 53 metastases), to the best of our knowledge, our study is the first of its kind focusing on the outcome of RT combined with AD for bone oligometastases after primary curative RT for PCa. However, there are several limitations in our study. First, this is a retrospective analysis with selection bias. Second, the limited number of patients is inadequate to establish the best modality for oligometastatic PCa. Finally, our study lacked a control group to determine the superiority of conventional EBRT over active clinical surveillance. However, an ongoing trial (clinicaltrials.gov identifier NCT01558427, University Hospital Ghent) compares salvage treatment of oligometastatic PCa with either surgery or RT for active clinical surveillance. Fifty-four patients who are not castrated and have a well-controlled primary tumor will be enrolled. The primary endpoint of the trial is ADT-free survival, and expected study completion date is May 2017. The underlying hypothesis is that patients under active surveillance have poor prognosis compared with those undergoing aggressive local treatments. Therefore, a larger prospective trial with controlled group is needed to estimate the actual benefit of RT combined with ADT for PCa bone oligometastases.

In conclusion, PCa patients diagnosed with 3 or less bone metastases after failure following curative RT of the local tumor may be successfully treated with long-course RT and AD. The number of bone oligometastases and RT schedule were found to be significantly associated with OS. This favorable correlation indicated, however, the potential role of RT combined with hormone therapy for bone oligometastases in PCa.
